# Calcium Fructoborate for Bone and Cardiovascular Health

**DOI:** 10.1007/s12011-015-0590-2

**Published:** 2015-12-21

**Authors:** George Dan Mogoşanu, Andrei Biţă, Ludovic Everard Bejenaru, Cornelia Bejenaru, Octavian Croitoru, Gabriela Rău, Otilia-Constantina Rogoveanu, Dan Nicolae Florescu, Johny Neamţu, Iulia Daria Scorei, Romulus Ion Scorei

**Affiliations:** Department of Pharmacognosy & Phytotherapy, Faculty of Pharmacy, University of Medicine and Pharmacy of Craiova, 2 Petru Rareş Street, 200349 Craiova, Romania; Department of Vegetal & Animal Biology, Faculty of Pharmacy, University of Medicine and Pharmacy of Craiova, 2 Petru Rareş Street, 200349 Craiova, Romania; Department of Drug Control, Faculty of Pharmacy, University of Medicine and Pharmacy of Craiova, 2 Petru Rareş Street, 200349 Craiova, Romania; Department of Organic Chemistry, Faculty of Pharmacy, University of Medicine and Pharmacy of Craiova, 2 Petru Rareş Street, 200349 Craiova, Romania; Department of Physical Medicine and Rehabilitation, Faculty of Medicine, University of Medicine and Pharmacy of Craiova, 2 Petru Rareş Street, 200349 Craiova, Romania; Research Center of Gastroenterology and Hepatology, University of Medicine and Pharmacy of Craiova, 2 Petru Rareş Street, 200349 Craiova, Romania; Department of Physics, Faculty of Pharmacy, University of Medicine and Pharmacy of Craiova, 2 Petru Rareş Street, 200349 Craiova, Romania; BioBoron Research Institute, Mirceşti Street, Bldg. M4/1/1, 200506 Craiova, Romania

**Keywords:** Calcium fructoborate, Sugar-borate esters, Anti-inflammatory activity, Cardiovascular health, Cytokines, Clinical studies

## Abstract

Calcium fructoborate (CF), a natural sugar-borate ester found in fresh fruits and vegetables, is a source of soluble boron. CF contains three forms of borate (diester, monoester, and boric acid) and all are biologically active, both at the intracellular (as free boric acid) and extracellular level (as fructose-borate diester and monoester). At the cellular and molecular level, CF is superior to the boric acid/borate, exhibiting a complex “protective” effect against inflammatory response. CF is commercially available in the USA as a “nature-identical” complex, an active compound for dietary supplements. It provides effective and safe support against the discomfort and lack of flexibility associated with osteoarticular conditions (arthritis and joint degeneration), and improves Western Ontario and McMaster Universities Osteoarthritis (WOMAC) and McGill indexes. In addition, orally administered CF is effective in ameliorating symptoms of physiological response to stress, including inflammation of the mucous membranes, discomfort associated with osteoarthritis disorders, and bone loss, and also for supporting cardiovascular health. Clinical studies have exhibited the ability of CF to significantly modulate molecular markers associated with inflammatory mechanisms, mainly on the elevated serum levels of C-reactive protein (CRP).

## Introduction

Calcium fructoborate (CF), a sugar-borate ester (SBE), is the most scientifically studied boron-based dietary supplement, with over a dozen of published studies on its unique chemical and clinical properties [1].

SBEs are found in fruits, vegetables, certain nuts, and legumes, and they are naturally absorbed by animal cells [2]. CF is the most common SBE, typically manifesting as the specific bis-fructose ester. In foods, they thus serve as a source of soluble borate. Currently, CF is manufactured as a “nature-identical” complex [3] and is commercially marketed as FruiteX-B® Brand calcium fructoborate (FrXB). Although calcium fructoborate is naturally occurring and found in commonly ingested fruits and vegetables, the commercially produced FrXB calcium fructoborate complex is formed by a proprietary reaction of boric acid with fructose and calcium carbonate [3]. FrXB is used as an active component of dietary supplement products in the USA for bone health and for modulation of the symptoms of arthritis and joint degeneration, justified by its reported clinical potency to reduce inflammation, and to improve Western Ontario and McMaster Universities Osteoarthritis (WOMAC) and McGill indexes [4, 5]. According to some recent clinical trials, FrXB can also be used to prevent cardiovascular disorders [6, 7].

## Intake, Occurrence, Chemistry, and Nutritional Significance of Calcium Fructoborate

### Intake and Occurrence

The bis-fructose ester of boric acid has been detected in plants, fruits, seeds, honey, and some foodstuff (Table 1); it is clear that fructoborate ester is a naturally occurring part of the human diet [Scorei et al., unpublished data]. Thus, the mean daily intake of fructoborate and related complexes is estimated to be about 35 mg (1.05 mg B), and the 95th percentile intake is estimated at about 75 mg (2.25 mg B) [1, 8]. In accordance with the “Dietary Reference Intakes”, in the USA boron-based compounds regulated as dietary supplements may provide up to 20 mg of elemental boron equivalents per day [9]. Recently, a simple, fast, specific, and precise high performance thin layer chromatography (HPTLC) and colorimetric methods have been developed and validated for the estimation of boron as boric acid, borax, and sugar-borate esters as calcium fructoborate esters (CFEs) in foodstuff and dietary supplements [10, 11].

**Table 1:** Content of total boron and fructoborate esters in various foodstuff (average values)

Food item	Total boron [ppm]	Fructoborate esters [ppm]
Dandelion root (Taraxaci radix)	200	80
Honey (mel)	12	7
Flaxseed sprouts (Lini semen)	800	80
Apple	25	3.5
Figs	35	15
Raisins	28	5
Tomato paste	20	7
Fructose	80	80

### Chemistry

The results of the thermal analysis, together with those of X-ray powder diffraction (XRD), Fourier Transform Infrared (FTIR) spectroscopy, and Raman spectroscopy, led to the conclusion that FrXB is a natural identical product with following molecular composition Ca[(C_6_H_10_O_6_)_2_B]_2_°4H_2_O, identical with the CF molecule found in nature, containing 2.5 ± 0.1 % boron and 4.6 ± 0.1 % calcium [12]. Other research has identified those three basic types of boron-containing molecules in aqueous solutions of FrXB: free boric acid, the diester complex, and the monoester complex. The relative molar concentrations of these three types of boron-containing molecules were found to be approximately 5, 85, and 10 %, respectively [13].

### Nutritional Significance

The significance of boron nutrition in the prevention of chronic disease has been recognized for some time in the global community [14, 15]. Symptoms of boron deficiency are non-specific, including arthritis, bone loss, decreased immunity, and osteoporosis [15–17]. In developing countries, borate supplementation may be effective for the prevention of osteoarthritis and osteoporosis [4, 5, 18], prostate [2, 19] and breast cancer [20], cardiovascular diseases [6], and recently, as an adjunct treatment for osteochondrosis in animals and humans [21]. Furthermore, boron was not found to be associated with the collagen matrix but almost entirely and exclusively located within the mineral portion of bone [22]. Boron is suggested to affect bone mineral by influencing serum steroid hormone levels (estrogen and testosterone) and the metabolism and utilization of calcium [23–25] and other mineral elements of bone [17, 25]. Furthermore, boron levels in bone and blood increasing with age and health state and decreased with disease conditions [1, 7, 8, 14, 18, 22, 25].

SBEs are regular components of the human diet found in vegetables and fruits that have previously been considered non-essential for human health. In the last decade, however, they have become the subject of intensive investigations because of their possible beneficial effects. Our results indicate that calcium fructoborate (the most common SBE), may exhibit a “protective” effect against inflammatory molecules at cellular and enzymatic levels [6, 7, 20, 26–28]. Calcium fructoborate, as a naturally occurring boron dietary derivative, serves as an important source of bioavailable dietary borate storage and, when administered orally, is effective in ameliorating symptoms of physiological response to stress, including inflammation of the mucous membranes, discomfort and stiffness associated with osteoarthritis disorders, and bone loss [8, 18].

## Clinical Studies

Most recently, published clinical researches have demonstrated CF’s ability to modulate key markers associated with the body’s inflammatory response mechanism. In particular, the studies indicate that CF significantly modulates elevated serum levels of C-reactive protein (CRP) in humans and some cytokines.

### Clinical Study No. 1

A double-blind, placebo-controlled study was performed on healthy volunteers, 10 subjects per group [4]. CF supplementation tested over a 14-day period at a serving of 108 mg (2.91 mg B and 5.4 mg Ca) twice per day [4]. CRP was reduced by 37 % versus baseline, pre-supplementation value. CF also induced a 19 % increase in endogenous levels of calcitriol, the active form of vitamin D_3_ (1,25-dihydroxyvitamin D). Blood level of CRP in 7 out of 10 subjects was found reduced up to 37 % compared to day 1 baseline levels. Interestingly, the study also showed that blood level of endogenous vitamin D_3_ was increased more than 19 % compared to baseline [4].

### Clinical Study No. 2

A double-blind, placebo-controlled study evaluated the effect of CF on systemic inflammation and dyslipidemia markers for middle-aged people with primary osteoarthritis, 15 subjects per group [29]. CF supplementation tested over a 14-day period at a serving of 57 mg per day (1.5 mg B and 2.85 mg Ca). CRP was reduced by 60.25 % versus baseline, pre-supplementation value. The CF group also experienced a reduction in elevated levels of erythrocyte sedimentation rate (−10.25 %) and fibrinogen (−13.73 %), other common measures of inflammation. This study also provides important information regarding the possible molecular mechanisms of an anti-inflammatory compound with an identical natural origin in the nutrition of osteoarthritis (OA) demonstrated that CF has potential efficacy in terms of reducing pain and improving the physical ability of OA patients. Summing up, the presented data have important implications for new strategy development for preventing OA and dyslipidemia associated with fructoborate supplementation [29].

### Clinical Study No. 3

A double-blind, active-controlled (existing treatment protocol) study has been achieved on patients with stable angina pectoris, 29 subjects per group [6]. CF supplementation tested over a 60-day period at a serving of 112 mg once per day (3 mg B and 5.26 mg Ca). CRP was reduced by 39.7 % versus baseline, pre-supplementation value. The CF group also experienced benefits on other clinical endpoints, including a 5.9 % reduction in total cholesterol, a 9.2 % reduction in LDL (low-density lipoprotein) cholesterol, a 5.1 % increase in HDL (high-density lipoprotein) cholesterol, and a 48.8 % reduction in angina episodes per week. In this study, high-sensitivity (hs)-CRP and pro-BNP (pro-hormone brain natriuretic peptide) showed significant changes in a relatively short time (2 months). This finding opens new directions of research regarding the use of natural adjuvants (CF plus resveratrol) for improving the standard anti-angina therapies [6].

### Clinical Study No. 4

A double-blind, placebo-controlled clinical study has been designed to determine the effects of CF on levels of CRP, total cholesterol (TC), LDL cholesterol, triglycerides, interleukin-1β (IL-1β), interleukin-6 (IL-6), and MCP-1 (monocyte chemoattractant protein-1) [7]. The objective of this study was to investigate whether CF alters blood levels of lipids, homocysteine, CRP, IL-1β, IL-6, and MCP-1 in generally healthy middle-aged subjects in order to evaluate the potential use of CF as a dietary supplement to support cardiovascular health. Our results suggest that use of CF at a daily dose as low as 112 mg for 30 days may significantly reduce levels of the pro-inflammatory and pro-atherogenic markers TC, LDL cholesterol, triglycerides, CRP, and homocysteine, while increasing the levels of HDL cholesterol, which is considered a protective lipid. Furthermore, supplemental use of CF at a dose of 112 mg per day (3 mg B and 5.26 mg Ca) appears to have a statistically significant inhibitory effect on pro-inflammatory cytokines such as IL-1β, IL-6, and MCP-1. In humans, plasma levels of MCP-1 correlate with the severity of cardiovascular conditions. Several researchers have postulated that blocking or reducing the expression of MCP-1 might be beneficial in preventing the development of unhealthy heart conditions [7].

### Clinical Study No. 5

A comparative, double-blind, placebo controlled acute clinical study has been designed to show the effects of calcium fructoborate short-term use in combination with chondroitin sulfate and glucosamine on improvement of knee discomfort conditions and physical mobility of the joints in healthy subjects [30]. There were three groups involved in the study of joint discomfort: the first one was treated with a blend of 750 mg glucosamine and 200 mg chondroitin sulfate, the second one was treated with a blend of 750 mg glucosamine, 200 mg chondroitin sulfate, and 110 mg calcium fructoborate (3 mg B and 5.4 mg Ca), and the third group was placebo (160 mg of fructose and 30 mg of silica oxide). Treatment with glucosamine combined with chondroitin sulfate and CF resulted in a statistically significant 24 % reduction of mean WOMAC score and a 25 % reduction of mean McGill index at day 14 over baseline (*p* = 0.0006 and *p* < 0.0001, respectively). Treatment with placebo or with glucosamine and chondroitin sulfate did not result in significant improvement of the conditions. The final result of this study clearly indicate that short-term use of CF in combination with chondroitin sulfate and glucosamine was effective in reducing knee discomfort and improving the joints’ physical mobility [30].

## Mechanism of Action of Calcium Fructoborate

It is known that at the cell pH of 7.4, boron (B) is connected with fructose only as free boric acid and fructoborate esters and not as anion borate (this is because fructoborates pK_a_ is 4.16) [18]. Overall, there are scientific data about a more general function for B, where B cross-links glycoproteins in cell membranes [31]. Subsequently, the most probable action mechanism of CF may be the chemical bonding of fructoborate to specific cytokines glycoproteic receptors at the surface of the cellular membrane. This may be happening since the fructoborate pK_a_ (4.16) is lower than the cellular pH (7.4), compared with the boric acid whose pK_a_ (9.24) is higher than the cellular pH. Consequently, fructoborate has a better interaction capacity with glycoproteins versus that of the boric acid/borate. Furthermore, the free boric acid generated by hydrolysis of the fructoborate complex was recently shown to crystallize in the hexagonal system rather than in the triclinic system as compared to the regular boric acid [32]. These different ways of crystallization may influence toxicity and biological activity of the free boric acid. This hypothesis is being currently investigated in our laboratory. Accordingly, the fructoborate complex is a non-toxic boron “reservoir” [33]. Our consideration is that fructoborate complex has physiological activity both within the cell (as free boric acid) and also outside of the cell (as fructoborates).

## Conclusions

The three-borate forms contained by the hydrolyzed calcium fructoborate (diester, monoester, and boric acid) are all biologically active, both at the intracellular (boric acid) and extracellular levels (diester and monoester). Transportation of the boric acid through the cellular membrane is being accomplished by free diffusion or is facilitated by aquaporin-like protein transporter. Recently, it has been reported that boron is not being transported as borate anion [34], but only as boric acid. If so, the exact mechanism of boron transportation in the animal cell remains unclear [35–37]. Consequently, CF is superior to the boric acid/borate due to its complex action mechanism, both at the intracellular (as free boric acid) and extracellular level (as fructose-borate esters). Moreover, the free boric acid resulting from hydrolysis of the fructoborate complex may be less toxic than the dietary supplement intake of the regular boric acid/borax/sodium borate. Because CF has been shown to be an efficient, non-toxic precursor of the borate anion, having multiple published studies on its numerous potential contributions to human health, nutritional supplementation with CF offers significant benefits in support of healthy bone and joints, as well as for cardiovascular health.
